# *In situ* detection of GM1 and GM2 gangliosides using immunohistochemical and immunofluorescent techniques for auxiliary diagnosis of canine and feline gangliosidoses

**DOI:** 10.1186/s12917-016-0691-y

**Published:** 2016-03-31

**Authors:** Moeko Kohyama, Akira Yabuki, Kenji Ochiai, Yuya Nakamoto, Kazuyuki Uchida, Daisuke Hasegawa, Kimimasa Takahashi, Hiroaki Kawaguchi, Masaya Tsuboi, Osamu Yamato

**Affiliations:** Laboratory of Clinical Pathology, Department of Veterinary Medicine, Joint Faculty of Veterinary Medicine, Kagoshima University, 1-21-24 Kohrimoto, Kagoshima-shi, Kagoshima, 890-0065 Japan; Laboratory of Veterinary Pathology, Department of Veterinary Medicine, Faculty of Agriculture, Iwate University, 3-18-8 Ueda, Morioka-shi, Iwate, 020-8550 Japan; Kyoto Animal Referral Medical Center, 208-4 Shin-arami, Tai, Kumiyama-cho, Kuse-gun, Kyoto, 613-0036 Japan; Laboratory of Veterinary Pathology, Graduate School of Agricultural and Life Sciences, The University of Tokyo, 1-1-1 Yayoi, Bunkyou-ku, Tokyo, 113-8657 Japan; Department of Veterinary Radiology, Nippon Veterinary and Life Science University, 1-7-1 Kyouman-chou, Musashino-shi, Tokyo, 180-8602 Japan; Department of Veterinary Pathology, Nippon Veterinary and Life Science University, 1-7-1 Kyouman-chou, Musashino-shi, Tokyo, 180-8602 Japan; Laboratory of Veterinary Histopathology, Department of Veterinary Medicine, Joint Faculty of Veterinary Medicine, Kagoshima University, 1-21-24 Kohrimoto, Kagoshima-shi, Kagoshima, 890-0065 Japan

**Keywords:** Gangliosidosis, Dog, Cat, Lysosomal Storage Disease, Immunohistochemistry, Immunofluorescence

## Abstract

**Background:**

GM1 and GM2 gangliosidoses are progressive neurodegenerative lysosomal storage diseases resulting from the excessive accumulation of GM1 and GM2 gangliosides in the lysosomes, respectively. The diagnosis of gangliosidosis is carried out based on comprehensive findings using various types of specimens for histological, ultrastructural, biochemical and genetic analyses. Therefore, the partial absence or lack of specimens might have resulted in many undiagnosed cases. The aim of the present study was to establish immunohistochemical and immunofluorescent techniques for the auxiliary diagnosis of canine and feline gangliosidoses, using paraffin-embedded brain specimens stored for a long period.

**Results:**

Using hematoxylin and eosin staining, cytoplasmic accumulation of pale to eosinophilic granular materials in swollen neurons was observed in animals previously diagnosed with GM1 or GM2 gangliosidosis. The immunohistochemical and immunofluorescent techniques developed in this study clearly demonstrated the accumulated material to be either GM1 or GM2 ganglioside.

**Conclusions:**

Immunohistochemical and immunofluorescent techniques using stored paraffin-embedded brain specimens are useful for the retrospective diagnosis of GM1 and GM2 gangliosidoses in dogs and cats.

## Background

GM1 and GM2 gangliosidoses are progressive neurodegenerative lysosomal storage diseases resulting mainly from the excessive accumulation of GM1 and GM2 gangliosides in the lysosomes, respectively [1]. These diseases are inherited in an autosomal recessive manner and result in the premature death of affected individuals due to brain damage with progressive neurological signs. In GM1 gangliosidosis, the accumulation of GM1 ganglioside is caused by an inherited deficiency of the lysosomal acid β-galactosidase [2]. In GM2 gangliosidosis, the accumulation of GM2 ganglioside is caused by an inherited deficiency of the lysosomal acid β-hexosaminidase A or GM2 activator protein in GM2 gangliosidosis, and the disease is accordingly categorized into three variants: Tay-Sachs disease (B variant), Sandhoff disease (0 variant), and GM2 activator protein deficiency (AB variant) [3].

Gangliosidosis is more likely to occur in many animal species and breeds compared to other lysosomal diseases. Naturally occurring GM1 gangliosidosis has been reported in dogs, including mixed Beagles [4], English Springer Spaniels [5], Portuguese Water dogs [6], Alaskan Huskies [7], Shiba Inus [8], and a mixed-breed dog [9], and in cats, including Siamese [10, 11], Korat [12], and several families of domestic cats [13–17]. In addition, GM1 gangliosidosis has been reported in ruminants such as Friesian calves [17, 18], Suffolk sheep [19], Coopworth Romny-cross sheep [20], and Romny sheep [21], and in wild species such as American black bears [22] and emus [23]. Naturally occurring GM2 gangliosidosis has been reported in dogs, including German Shorthair Pointers [24], Japanese Spaniels (Chins) [25, 26], a Golden Retriever [27], Toy Poodles [28], and mixed-breed dogs [29, 30], and in cats, including Korat [31], European Burmese [32], and several families of domestic cats [33–35]. In addition, GM2 gangliosidosis has been reported in Yorkshire pigs [36], Jacob sheep [37], a rabbit [38], Muntjak deer [39], and American flamingos [40].

The diagnosis of GM1 and GM2 gangliosidoses is carried out based on comprehensive findings, which include clinical, biochemical, histopathological, and genetic findings using various types of specimens [2, 3]. The clinical findings are progressive neurological, motor, and visual dysfunctions, but they are not specific to these diseases [41]. The biochemical findings include the cerebral accumulation of specific glycoconjugates and deficiency of specific enzyme activities, which are determined by specialized techniques such as thin-layer chromatography (TLC) and fluorometric enzymatic assays, respectively, using fresh or frozen tissues [42, 43]. The histopathological and ultrastructural findings demonstrate swollen neurons filled with periodic acid-Schiff stain-positive storage materials and osmiophilic membranous cytoplasmic bodies in the lysosomes of neurons, respectively, but these characteristics are not completely specific to these diseases [8, 28, 30, 34]. Genetic tests can be used to directly diagnose the diseases, but they are limited to diseases for which specific mutations have been identified [44–46]. Therefore, it is possible that a correct diagnosis has not been established in many animal cases, as a result of the partial absence or lack of specimens for biochemical, histological, ultrastructural, or genetic examination.

The aim of the present study was to establish immunohistochemical and immunofluorescent techniques for the auxiliary diagnosis of canine and feline gangliosidoses using paraffin-embedded brain specimens, which are often stored for a long time in veterinary diagnostic laboratories worldwide.

## Methods

### Specimens

Stored paraffin-embedded cerebral cortex samples of dogs and cats with GM1 or GM2 gangliosidosis were used in this study. These cases occurred in different parts of Japan and the original diagnosis was made using specific genetic tests and biochemical analyses at the Laboratory of Clinical Pathology, Joint Faculty of Veterinary Medicine, Kagoshima University, which has been exclusively supporting the diagnosis of inherited metabolic diseases in animals in Japan. These animals included a 14-month-old Shiba Inu with GM1 gangliosidosis diagnosed in 2009, an 11-month-old domestic shorthair cat with GM1 gangliosidosis diagnosed in 2004, a 20-month-old Toy Poodle with GM2 gangliosidosis diagnosed in 2006, and a 20-month-old domestic shorthair cat with GM2 gangliosidosis diagnosed in 2010. The diagnosis of these animals was established using genetic and/or biochemical tests reported previously [11, 43–45]. Stored paraffin-embedded cerebral cortex samples of a dog and a cat without any brain disease were also used as controls. Thin sections at 4 μm were prepared from these paraffin-embedded tissue blocks by standard method. These sections were stained with hematoxylin and eosin (HE) and subjected to the immunohistochemical and immunofluorescent techniques described below. All experimental procedures and ethical issues involving animals and their samples were approved by the the Animal Research Committee at Kagoshima University with the approval number VM15041.

### Immunohistochemical study

Each section was deparaffinized with xylene and rehydrated through a graded ethanol series. Antigen retrieval was conducted by heating the sample in a 10 mM citrate buffer (pH 6.0) in a microwave oven. Thereafter, the samples were washed in deionized water, treated with 3 % hydrogen peroxide, and washed in 0.01 M phosphate-buffered saline (PBS; pH 7.4). Blocking was performed with 0.25 % casein in 0.01 M PBS and incubated overnight at 4°C with the respective reagents.

For the detection of GM1 ganglioside, biotinylated cholera toxin B subunit (1:1000; List Biological Laboratories, Inc., Campbell, CA, USA) was used. For the detection of GM2 ganglioside, mouse anti-GM2 ganglioside monoclonal IgM antibody (1:1000; Tokyo Chemical Industry, Co., Ltd., Tokyo, Japan) was used as a primary antibody, and biotinylated goat anti-mouse IgM antibody (1:200; Vector Laboratories, Inc., Burlingame, CA, USA) was used as a secondary antibody. Subsequently, these sections were incubated with peroxidase-labeled streptavidin (KPL, Kirkegaard & Perry Laboratories, Inc., Gaithersburg, MD, USA). The immunoreactivity was detected by a 3,3′-diaminobenzidine (DAB) system using DAB Tablet (Merck KGaA, Darmstadt, Germany) as a peroxidase substrate. The sections were counterstained with hematoxylin.

### Immunofluorescent study

Each section was pretreated in the same way as described above for immunohistochemistry. For the detection of GM1 ganglioside, biotinylated cholera toxin B subunit (1:500; List Biological Laboratories, Inc.) and Alexa Fluor 488-conjugated streptavidin (1:1000; Life Technologies, Inc., Gaithersburg, MD, USA) were used. For the detection of GM2 ganglioside, mouse anti-GM2 monoclonal IgM antibody (1:500; Vector Laboratories, Inc.) was used as a primary antibody, and Alexa Fluor 488-conjugated goat anti-mouse IgM antibody (1:1000; Life Technologies, Inc.) was used as a secondary antibody. Subsequently, these sections were incubated with a 4′,6-diamidino-2-phenylindole dihydrochloride (DAPI) solution (1:1000; Dojindo Laboratories, Inc., Kumamoto, Japan) for nuclear staining. The fluorescence was observed using a fluorescence microscopy (BX53-33-FL2, Olympus, Corp., Tokyo, Japan).

## Results

**Fig. 1:**
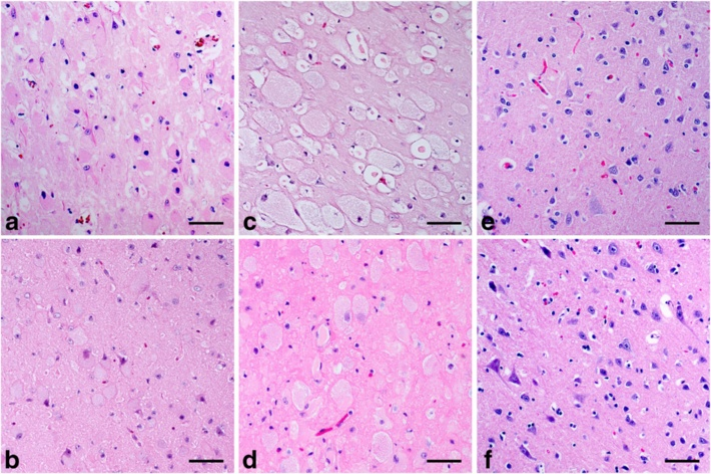
Histopathological findings in animals affected and unaffected with gangliosidoses. Hematoxylin and eosin staining was performed on paraffin-embedded sections of the cerebral cortex from the following animals: a dog (**a**) and a cat (**b**) affected with GM1 gangliosidosis; a dog (**c**) and a cat (**d**) affected with GM2 gangliosidosis; an unaffected control dog (**e**) and cat (**f**). Bar = 50 μm

Using the HE stain, cytoplasmic accumulation of pale to eosinophilic granular materials in balloon-swollen neurons was observed in the cerebral cortex samples of dogs and cats previously diagnosed with GM1 or GM2 gangliosidosis (Fig. 1a–d), whereas there was no such abnormal change observed in the samples of the control animals (Fig. 1e and f).

**Fig. 2:**
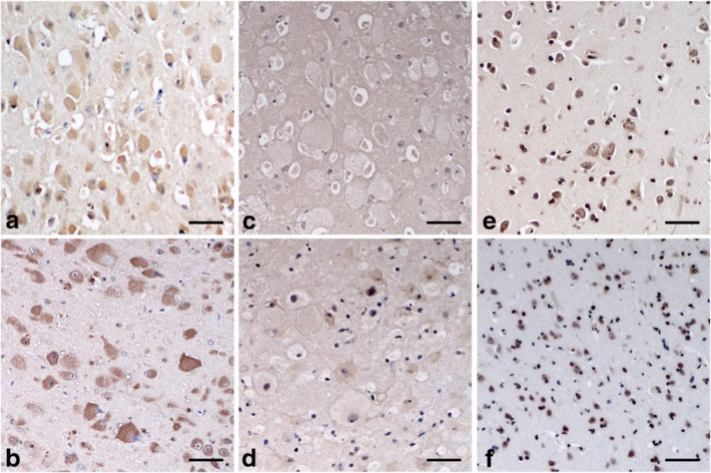
Immunohistochemical findings for the detection of GM1 ganglioside in animals affected and unaffected with gangliosidoses. The immunohistochemical technique for the detection of GM1 ganglioside was performed on paraffin-embedded sections of the cerebral cortex from the following animals: a dog (**a**) and a cat (**b**) affected with GM1 gangliosidosis; a dog (**c**) and a cat (**d**) affected with GM2 gangliosidosis; an unaffected control dog (**e**) and cat (**f**). For the detection of GM1 ganglioside, biotinylated cholera toxin B subunit and peroxidase-labeled streptavidin were used. The immunoreactivity was detected by 3,3′-diaminobenzidine as a peroxidase substrate. The sections were counterstained with hematoxylin. Bar = 50 μm

Using the immunohistochemical technique for the detection of GM1 ganglioside, the accumulated cytoplasmic materials were positively stained and mainly identified as GM1 ganglioside in cells of animals with confirmed GM1 gangliosidosis (Fig. 2a and b). In animals with GM2 gangliosidosis, the accumulated cytoplasmic materials were very weakly positively stained in a portion of the cells of the affected cat (Fig. 2c and d). In the control animals, the cytoplasm in some normal-shaped cells was also positively stained to indicate the presence of GM1 ganglioside (Fig. 2e and f). The nuclei of several cells were positively stained in a portion of the samples such as in the case of feline GM2 gangliosidosis and in both control animals (Fig. 2d–f).

**Fig. 3:**
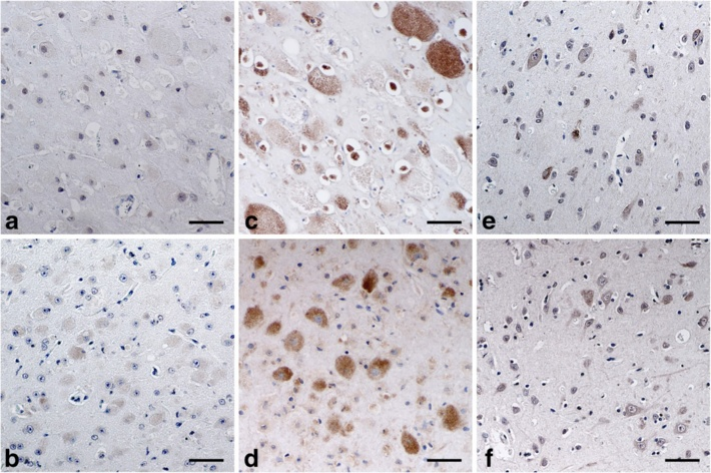
Immunohistochemical findings for the detection of GM2 ganglioside in animals affected and unaffected with gangliosidoses. The immunohistochemical technique for the detection of GM2 ganglioside was performed on paraffin-embedded sections of the cerebral cortex from the following animals: a dog (**a**) and a cat (**b**) affected with GM1 gangliosidosis; a dog (**c**) and a cat (**d**) affected with GM2 gangliosidosis; an unaffected control dog (**e**) and cat (**f**). For the detection of GM2 ganglioside, mouse anti-GM2 ganglioside monocloncal IgM antibody was used as a primary antibody, and biotinylated goat anti-mouse IgM antibody was used as a secondary antibody. Subsequently, these sections were incubated with peroxidase-labeled streptavidin. The immunoreactivity was detected by 3,3′-diaminobenzidine as a peroxidase substrate. The sections were counterstained with hematoxylin. Bar = 50 μm

Using the immunohistochemical technique for the detection of GM2 ganglioside, the accumulated cytoplasmic materials were positively stained and mainly identified as GM2 ganglioside in the cells of animals with GM2 gangliosidosis (Fig. 3c and d), whereas these materials were not strongly stained in animals with GM1 gangliosidosis (Fig. 3a and b). In the control animals, the cytoplasm in some normal-shaped cells was also weakly stained using this method (Fig. 3e and f). The nuclei of several cells were weakly positively stained in a portion of the samples such as in the case of canine GM1 gangliosidosis and in both control animals (Fig. 3a, e and f).

**Fig. 4:**
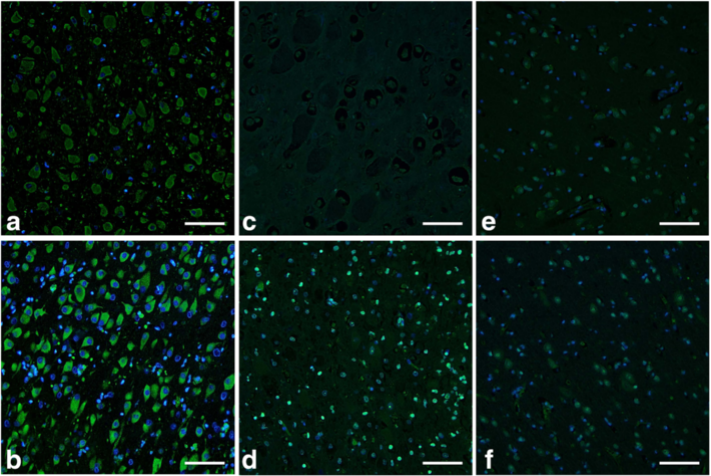
Immunofluorescent findings for the detection of GM1 ganglioside in animals affected and unaffected with gangliosidoses. The immunofluorescent technique for the detection of GM1 ganglioside was performed on paraffin-embedded sections of the cerebral cortex from the following animals: a dog (**a**) and a cat (**b**) affected with GM1 gangliosidosis; a dog (**c**) and a cat (**d**) affected with GM2 gangliosidosis; an unaffected control dog (**e**) and cat (**f**). For the detection of GM1 ganglioside, biotinylated cholera toxin B subunit and Alexa Fluor 488-conjugated streptavidin were used. Subsequently, these sections were incubated with 4′,6-diamidino-2-phenylindole dihydrochloride for nuclear staining. Bar = 30 μm

**Fig. 5:**
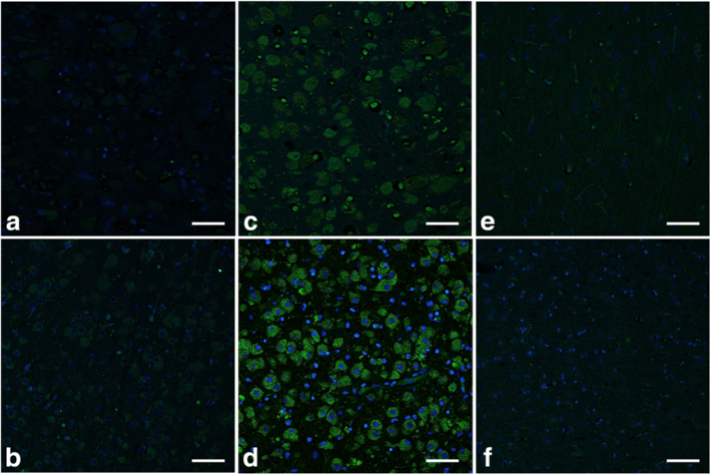
Immunofluorescent findings for the detection of GM2 ganglioside in animals affected and unaffected with gangliosidoses. The immunofluorescent technique for the detection of GM2 ganglioside was performed on paraffin-embedded sections of the cerebral cortex from the following animals: a dog (**a**) and a cat (**b**) affected with GM1 gangliosidosis; a dog (**c**) and a cat (**d**) affected with GM2 gangliosidosis; an unaffected control dog (**e**) and cat (**f**). For the detection of GM2 ganglioside, mouse anti-GM2 monoclonal IgM antibody was used as a primary antibody, and Alexa Fluor 488-conjugated goat anti-mouse IgM antibody was used as a secondary antibody. Subsequently, these sections were incubated with 4′,6-diamidino-2-phenylindole dihydrochloride for nuclear staining. Bar = 30 μm

The results of the immunofluorescent technique were almost identical to those of the immunohistochemical technique. The accumulated materials in the swollen neurons of animals with gangliosidoses were positively stained and clearly identified as either GM1 or GM2 ganglioside by using the respective detection techniques for each ganglioside (Figs. 4 and 5). The accumulated materials in the neurons of animals with GM2 gangliosidosis were very weakly stained using the technique for GM1 ganglioside (Fig. 4c and d), and vice versa (Fig. 5a and b). In the control animals, some cells showed cytoplasm that was positively stained for GM1 and GM2 gangliosides (Figs. 4 and 5e and f). In addition, some of the cells of a cat with GM2 gangliosidosis and both control animals showed positive staining of nuclei using the technique for the detection of GM1 ganglioside (Fig. 4d–f). Some of the cells of a control cat showed weakly positive staining of nuclei using the technique for the detection of GM2 ganglioside (Fig. 5f).

## Discussion

Gangliosides are glycosphingolipids consisting of a hydrophobic ceramide (*N*-acylsphingosine) and a hydrophilic oligosaccharide chain bearing one or more *N*-acetylneuraminic acid (silalic acid) residues, and are typical components of the outer leaflet of the plasma membranes of animal cells [2, 3]. GM1 and GM2 gangliosides are present as the main glycolipids in neurons and are likely to be involved in cell differentiation and cell–cell interactions, but their specific physiological functions remain obscure. Therefore, developing techniques for the detection of GM1 and GM2 gangliosides is important not only for advancement in brain science but also for the correct diagnosis of gangliosidoses, because the intralysosomal accumulation of each ganglioside in neurons is characteristic to either GM1 or GM2 gangliosidosis. Therefore, in the past few decades, various determination methods for the profiling, quantification, or evaluation of gangliosides, including GM1 and GM2 gangliosides, in tissues, cultured cells, or extracellular fluids have been reported. These methods include TLC coupled with densitometric or immunochemical detection [43, 47], high-performance liquid chromatography coupled with tandem mass spectrometric detection [48], enzyme-linked immunosorbent assay [49], and matrix-assisted laser desorption ionization time-of-flight mass spectrometry [50].

The *in situ* detection of gangliosides in tissue sections is also very important not only for diagnosis of the diseases but also to obtain reliable information on their tissue, cellular, and subcellular distributions [51]. Furthermore, confirming that the histological detection of GM1 and GM2 gangliosides is applicable to paraffin-embedded specimens stored for a long period would also be useful for the retrospective diagnosis of the diseases, but very few studies have evaluated such *in situ* detection methods using long-term stored paraffin-embedded specimens from canine and feline gangliosidoses. In the present study, immunohistochemical and immunofluorescent techniques for the detection of GM1 and GM2 gangliosides were developed, and their application was evaluated using canine and feline paraffin-embedded specimens stored for 5 to 11 years. As a result, these two techniques could clearly detect the presence of both GM1 and GM2 gangliosides in neurons of the control animals (Figs. 2, 3, 4 and 5e and f) as well as the accumulation of either GM1 or GM2 ganglioside in neurons of animals with diagnosed GM1 (Figs. 2, 3, 4 and 5a and b) and GM2 gangliosidoses (Figs. 2, 3, 4 and 5c and d). These data demonstrate that the two techniques are applicable to the retrospective *in situ* detection of GM1 and GM2 gangliosides, and consequently to the auxiliary diagnosis of gangliosidoses in dogs and cats. However, gangliosides can be accumulated as the secondary products without direct link to the primary protein defect in some lysosomal and a few non-lysosomal diseases [2, 52]. Therefore, in cases in which the abnormal accumulation of each ganglioside is found in swollen neurons, a definitive diagnosis should ultimately be made using DNA extracted from the same paraffin-embedded specimen via the identification of pathogenic mutation(s) in the responsible genes: the *GLB1* gene for GM1 gangliosidosis and the *HEXA*, *HEXB*, and *GM2A* genes for GM2 gangliosidosis.

Comparing the two techniques developed in the present study, the immunofluorescent technique provided relatively less histopathological information than the immunohistochemical technique, due to the dark background when using immunofluorescence. Therefore, the tissue, cellular, and subcellular distributions of stained materials could not be easily determined in the immunofluorescent technique; however, this technique does have the advantage of requiring a lower amount of reagents (nearly half) because of its higher detection sensitivity compared to the immunohistochemical technique.

In addition, in the experiments conducted to detect GM1 and GM2 gangliosides, the nuclei were stained in some specimens using both techniques. The positive staining of the nuclei in some cells from affected and control animals may result from the natural components of GM1 and GM2 gangliosides because the nuclei of neuronal cells in rat brain contain these gangliosides [53]. However, this stain of the nucleus was easily differentiated from the specific stain of cytoplasmic GM1 and GM2 gangliosides when using the immunohistochemical technique but not when using the immunofluorescent technique, owing to the reduced morphological visibility. Therefore, the simultaneous observation of HE-stained cerebral tissues (Fig. 1) is necessary for accurate judgment of the results, especially when using an immunofluorescent technique.

## Conclusions

The immunohistochemical and immunofluorescent techniques for the detection of GM1 and GM2 gangliosides established in this study are useful for the auxiliary diagnosis of GM1 and GM2 gangliosidoses in dogs and cats before a definitive diagnosis can be made using molecular analysis for identification of causative mutations. These techniques may also be useful for the retrospective diagnosis of suspected cases of all animal species for which paraffin-embedded cerebral tissues are stored.

## Abbreviations

DAB: 3,3′-diaminobenzidine
DAPI: 4′,6-diamidino-2-phenylindole dihydrochloride
HE: hematoxylin and eosin
PBS: phosphate-buffered saline
TLC: thin-layer chromatography
